# The disorganized visual cortex in reelin-deficient mice is functional and allows for enhanced plasticity

**DOI:** 10.1007/s00429-014-0866-x

**Published:** 2014-08-15

**Authors:** Justyna Pielecka-Fortuna, Robin Jan Wagener, Ann-Kristin Martens, Bianka Goetze, Karl-Friedrich Schmidt, Jochen F. Staiger, Siegrid Löwel

**Affiliations:** 1 Department of Systems Neuroscience, Bernstein Fokus Neurotechnologie, Johann-Friedrich-Blumenbach-Institut für Zoologie und Anthropologie, Georg-August-Universität Göttingen, Von-Siebold-Str. 6, 37075 Göttingen, Germany; 2 Institute for Neuroanatomy, Universitätsmedizin Göttingen, Georg-August-Universität Göttingen, Kreuzbergring 36, 37075 Göttingen, Germany; 3 Center Nanoscale Microscopy and Molecular Physiology of the Brain (CNMPB), Göttingen, Germany; 4 Collaborative Research Center 889, University of Göttingen, 37075 Göttingen, Germany

**Keywords:** Primary visual cortex, Cortical plasticity, *Reeler*, Laminar fate, Thalamocortical

## Abstract

A hallmark of neocortical circuits is the segregation of processing streams into six distinct layers. The importance of this layered organization for cortical processing and plasticity is little understood. We investigated the structure, function and plasticity of primary visual cortex (V1) of adult mice deficient for the glycoprotein reelin and their wild-type littermates. In V1 of *rl*−/− mice, cells with different laminar fates are present at all cortical depths. Surprisingly, the (vertically) disorganized cortex maintains a precise retinotopic (horizontal) organization. *Rl*−/− mice have normal basic visual capabilities, but are compromised in more challenging perceptual tasks, such as orientation discrimination. Additionally, *rl*−/− animals learn and memorize a visual task as well as their wild-type littermates. Interestingly, reelin deficiency enhances visual cortical plasticity: juvenile-like ocular dominance plasticity is preserved into late adulthood. The present data offer an important insight into the capabilities of a disorganized cortical system to maintain basic functional properties.

## Introduction

The primary visual cortex (V1) as all other primary sensory areas of the neocortex is organized into six morphologically distinct laminae. Each of the different cortical layers is populated with a characteristic set of neuronal cell types and connections with other cortical and/or subcortical regions. This highly elaborated arrangement is thought to be crucial for proper function of neuronal networks (Douglas and Martin 2007; Markov et al. 2013). One of the molecules that have been shown to be vital for the proper establishment of laminar organization is a large extracellular matrix glycoprotein called reelin, secreted by the Cajal–Retzius cells in the marginal zone of the cortex (Caviness 1982; D’Arcangelo et al. 1995). The reelin gene autosomal recessive mutation was first discovered in 1951 and due to its reeling gait the mutant mouse strain was called *reeler* (Falconer 1951). The *reeler* (*rl*−/−*)* brain shows cerebellar hypotrophy and severe cellular disorganization of laminated brain structures, but all anatomical structures are in the correct general spatial position (Caviness and Sidman 1973; Goffinet 1984a). Based on the results of various birth-dating experiments, it was commonly believed that, roughly speaking, the *rl*−/− cortex is inverted (Caviness and Sidman 1973). Taking advantage of lamina-specific mRNA markers (Lein et al. 2007), we were recently able to demonstrate a much more severe defect in the structural organization of the primary somatosensory area of *rl*−/− mice (Wagener et al. 2010): neuronal populations destined for particular cortical layers were intermingled and spread over the whole cortical thickness. Moreover, a recent study strongly suggested the presence of distinct patterns of disorganization in different cortical areas, thus also implying a non-uniform reelin function in the different areas (Boyle et al. 2011). Despite these laminar defects, columnar modules in *rl*−/− S1 were arranged in an orderly somatotopic map (Wagener et al. 2010; Guy et al. 2014). Likewise, initial electrophysiological and anatomical studies have suggested that neurons in visual cortical areas of *rl*−/− mice are capable of forming retinotopically organized corticocortical and thalamocortical connections, in a pattern similar to that found in normal animals (Drager 1981; Simmons et al. 1982; Simmons and Pearlman 1982). However, detailed information about visual cortical lamination, structural and functional maps and their plasticity in *rl*−/− mice is still missing.

In the mature brain, reelin is expressed by a variety of GABAergic interneurons distributed throughout neocortical and hippocampal layers (Alcantara et al. 1998; Ramos-Moreno et al. 2006), which have been implicated in the promotion of learning mechanisms (Herz and Chen 2006). Reelin deficiency was shown to alter cortico-striatal plasticity, most likely by reducing numbers of parvalbumin positive (PV^+^) neurons in the striatum (Marrone et al. 2006). PV^+^ GABAergic neurons and perineuronal nets (PNNs) play an important role for visual cortex plasticity (Pizzorusso et al. 2002; Hensch 2005; Galtrey and Fawcett 2007; de Vivo et al. 2013), and their respective pharmacological manipulation was shown to promote experience-dependent V1 plasticity (Kirkwood et al. 1995; Hensch et al. 1998; Huang et al. 1999; Pizzorusso et al. 2002; Galtrey and Fawcett 2007).

To understand the implications of reelin deficiency for development, function and plasticity of V1, it is of high scientific interest to determine (i) the precise cellular organization defect, and how this affects (ii) the detailed functional organization of *rl*−/− V1, (iii) experience-dependent plasticity of its circuits and (iv) more elaborate visual capabilities. Utilizing state-of-the-art anatomical techniques, in vivo optical imaging of intrinsic signals and behavioral vision tests our data offer an astonishing insight into the capabilities of a disorganized cortical system to maintain basic functional properties and additionally show a new and unexpected role of reelin in modulating cortical plasticity.

## Materials and methods

*Animals* Sixty adult B6C3Fe mice (30 WT and 30 *rl*−/−) and 6 LIV tdTomato (3 WT and 3 *rl*−/−) were used for the experiments. Mice were obtained from the colony of the central animal facility of the University Medicine Göttingen and were housed on 12-h light/dark cycle, with food and water available ad libitum. All experimental procedures were approved by the local government.

*Fixation and tissue processing* For in situ hybridization and immunohistochemistry adult animals (>90d) were perfused transcardially with 10 ml of 10 % sucrose, followed by 200 ml of 4 % paraformaldehyde in 0.1 M phosphate buffer (PB), pH 7.4. Brains were dissected out and postfixed for 2 h. They were then cryoprotected in 20 % sucrose in 0.01 M phosphate buffered saline (PBS) overnight at 4 °C and sectioned in the standard coronal plane with a cryostat (Leica) at 40 µm thickness.

*In situ* *hybridization* (ISH) ISH for the layer-specific markers *Ndnf* (also known as *A930038C07Rik)*, *Rgs8*, *Rorb* (also known as *Rorbeta*), *Etv1* (also known as *Er81*) and *Foxp2* was performed with digoxigenin (DIG)-labeled cRNA probes in four brains (2 WT, 2 *rl*−/−). The probes were generated from the appropriate plasmids (Source BioScience) containing full-length cDNA inserts specific for the respective laminar marker. NCBI Gene IDs: *Ndnf* (68169), *Rgs8* (67792), *Rorb* (225998), *Etv1* (14009), *Foxp2* (114142). After restriction digestions with the appropriate enzymes and subsequent in vitro transcription using a DIG RNA labeling kit (Roche), the size of the probes was reduced to 350 base pairs via alkaline hydrolysis (0.2 M sodium carbonate and 0.2 M sodium bicarbonate at pH 10.2). The frozen brain sections were rinsed three times with 2× standard saline citrate (1 × SSC = 0.15 M NaCl, 0.015 M sodium citrate, pH 7.0) and pretreated for 15 min in hybridization buffer (HB; 50 % formamide, 4 × SSC, 250 µg/ml denaturated salmon sperm DNA, 100 µg/ml tRNA, 5 % dextransulfate and 1 % Denhardt’s solution) diluted 1:1 in 2 × SSC at room temperature, followed by 1 h in pure HB at 55 °C. Probe hybridization with DIG-labeled probes (200 ng/ml) was done overnight at the same temperature. Post-hybridization washes were performed with 2 × SSC (2 × 15 min, at room temperature), 2 × SSC/50 % formamide (15 min, at 65 °C), 0.1 × SSC/50 % formamide (15 min, at 65 °C), 0.1 × SSC (2 × 15 min, at 65 °C) and 0.01 M Tris-buffered saline, pH 7.4 (TBS; 2 × 10 min, at room temperature). The probes were detected by anti-DIG Fab fragments conjugated to alkaline phosphatase (raised in sheep; Roche) diluted 1:1,500 in TBS containing blocking agent (at 4 °C overnight). Hybridized probes were stained with nitroblue tetrazolium and 5-bromo-4-chloro-3-indolylphosphate (Roche). Development of the staining reaction was monitored with a light microscope (Zeiss). After the desired intensity was reached, the sections were rinsed again in TBS and mounted in Kaiser’s glycerol gelatin (Merck).

*Transgenic animals and stereotactically targeted viral tracer injection* Scnn1a-Cre mice (full strain name: B6.Cg-Tg(Scnn1a-cre)3Aibs/J; Stock Number: 009613; Jackson Laboratory) were crossed with heterozygous *reeler* animals (B6C3Fe *Reln* ±). The resulting Scnn1a-Cre *reeler (*±*)* were crossed again and animals homozygous for Scnn1a-Cre and heterozygous for *Reln* were chosen for further crossings. ROSA-Tomato-LSL mice (full strain name: B6;129S6-Gt(ROSA)26Sortm9(CAG-tdTomato)Hze/J; Stock number:007905; Jackson Laboratory) were crossed with heterozygous *reeler* in the same way; the resulting strain was called ROSA-Tomato-LSL *reeler* (±). Crossing of Scnn1a-Tg3-Cre *reeler* (±) and Tomato-LSL *reeler* (±) resulted in layer IV-specific expression of tdTomato. Thus, the respective line was called layer IV tdTomato, either WT or *reeler* (Guy et al. 2014). Heterozygous *reeler* animals were not used for analysis. 3 WT and three *rl*−/− animals were used for the tracing experiment.

For anterograde tracing we used a Synaptophysin (Syp) expressing AAV1 vector. Syp was expressed under the Synapsin promoter and tagged with enhanced yellow fluorescent protein (eYFP). The viral vector was custom manufactured by the Penn Vector Core (University of Pennsylvania). For targeting, the animals were placed into a stereotactic frame (David Kopf Instruments). Two hundred nanoliter of the viral vector in sterile PBS was injected into the dorsolateral geniculate nucleus of the thalamus (dLGN; anterior–posterior: Bregma −2.30 mm; medial–lateral: ±2.08 mm; dorsal–ventral: −2,83 mm) via a Pressure System IIe (TooheySpritzer). The animals were kept alive for 12 days to allow for expression of the YFP-tagged Syp (perfusion and tissue processing see above). The native fluorescence signals were amplified via immunocytochemistry using a goat anti-GFP antibody (Abcam; 1:2000) to enhance the YFP signal (secondary antibody: donkey anti-goat-Alexa 488; Invitrogen), and a rabbit anti-RFP (Rockland; 1:500) to enhance the tdTomato Signal (secondary antibody: donkey anti-rabbit-Alexa 546; Invitrogen; for immuno-protocol see below).

*Parvalbumin immunocytochemistry combined with WFA* *histochemistry* The sections were rinsed in PB, TBS and TBS containing 0.4 % Triton X-100 (TBST; 20 min each). They were blocked with bovine serum albumin (Roth) containing 10 % normal goat serum (in TBST) for 90 min. The primary antibody was a polyclonal antiserum against parvalbumin (1:10,000; raised in rabbit; Swant), which was simultaneously incubated with a biotin-conjugated wisteria floribunda agglutinin lectin (WFA; Sigma) diluted 1:2,000 in TBST for 60 h at 6 °C. Afterward, sections were rinsed in TBST (4 × 15 min each). Incubation of the secondary antibody was conducted for 4 h at room temperature with an Alexa 488-conjugated anti-rabbit antibody (raised in goat; Invitrogen) diluted 1:500 in TBST. The biotin-WFA was simultaneously coupled to a streptavidin Alexa 594 conjugate (1:300; Invitrogen). After rinsing in TBST and TBS, DAPI staining was performed according to the manufacturer’s protocol (Molecular Probes). Sections were rinsed extensively before being mounted in Aqua Poly-Mount (Polysciences, Inc.).

### Behavioral vision tests

*Optomotry* Using the virtual-reality optomotor system developed by Prusky et al. (2004) we measured visual acuity, contrast sensitivity and temporal frequency thresholds of the optomotor reflex of *rl*−/− and WT mice. Briefly, freely moving animals were exposed to moving sine wave gratings (with a drift speed of 12°/s) of various spatial frequencies and contrasts, which they reflexively track by head movements as long as they can see the gratings. Spatial frequency at full contrast and contrast at six different spatial frequencies [0.031, 0.064, 0.092, 0.103, 0.192, 0.272 cycles/degree (cyc/deg)] were varied by the experimenter until the threshold of tracking was determined. Contrast sensitivity thresholds of the optomotor reflex measured in percent were converted into Michelson contrasts. For measuring temporal frequency thresholds, drift speed of the gratings (at 100 % contrast) was increased until tracking ceased. For technical reasons, drift speeds above 50°/s could not be tested. Measurements were performed at the following spatial frequencies (in cyc/deg): 0.064, 0.103, 0.150, 0.192, 0.272 and 0.331 and 0.400. Finally, visual acuity thresholds of the optomotor reflex were determined through the non-deprived (left) eye of monocularly deprived mice and through the left eye of non-deprived control mice: measurements were performed daily during the 7 days of deprivation and on the day when their visual cortical activity was analyzed by optical imaging of intrinsic signals.

*Visual water task (VWT)* Orientation discrimination was measured using the VWT, a visual discrimination task that is based on reinforcement learning (Prusky et al. 2000; Prusky and Douglas 2004). Briefly, mice were trained to distinguish horizontal from vertical square wave gratings of a low spatial frequency (0.086 cyc/deg, training phase) and then their ability to recognize increasingly smaller orientation differences was tested (test phase). In the training phase, the mice learned to swim towards a horizontal grating under which an escape platform was located (reward). Once 90 % accuracy was achieved, the test phase was started and the discrimination threshold of individual mice was determined by decreasing the orientation contrast of the two gratings until performance fell below 70 % accuracy. The smallest orientation contrast at which 70 % accuracy was achieved was taken as the minimum discrimination threshold. The position of the rewarded grating was alternated in a pseudorandom sequence over the training and test trials. Mice were tested as a group in sessions of 5–10 interleaved trials, with each session lasting ~45–60 min. No more than two sessions, separated by at least 1.5 h, were performed per day. Additionally, we checked 1 and 2 months later whether both WT and *rl*−/− mice remembered the learned task, i.e. swam towards the initially learned grating, using an orthogonally oriented grating as a distractor.

### Monocular deprivation (MD)

To challenge plasticity mechanisms, we deprived the right eye of vision for 7 days, according to published protocols (Gordon and Stryker 1996; Cang et al. 2005; Lehmann and Löwel 2008). Briefly, mice were anesthetized with 2 % isoflurane in 1:1 O_2_:N_2_O, lid margins were trimmed and an antibiotic gel (gentamicin gel) was applied. The eye was closed with two mattress sutures, and mice were put back to their cages. Animals were checked daily to make sure that the eyes remained closed. The age range of the mice was between postnatal day (P) 61 and 148. For optical imaging experiments, WT and *rl*−/− mice with MD were split into two groups of the average age of P80 and P140. Age range of WT mice without MD varied from P61 to 157 days and from P78 to 148 days for *rl*−/−; since there was no statistical difference in OD values between different ages (*p* > 0.05) data were pooled for display. The indicated age of mice is at the day of the terminal optical imaging experiment.

*Diazepam treatment* Four *rl*−/− (P147-186) and four WT mice (P90-93) were treated with diazepam (Rotexmedica, i.p.), an allosteric GABA_A_ receptor modulator, to increase GABAergic inhibition during MD. Injections started 3–4 h before MD and continued for 6 days with one injection per day. The dosage used (1 mg/kg) was selected as it did not affect normal activity and exploring behavior of the treated mice and was previously shown to reliably block OD plasticity in adult C57Bl/6J mice (Greifzu et al. 2014).

### Optical imaging of intrinsic signals and visual stimuli

*Surgery* Briefly, mice were initially box-anesthetized with 2 % halothane in a mixture of O_2_:N_2_O (1:1) and received an injection of atropine (Franz Köhler, 0.3 mg/mouse, subcutaneously), dexamethasone (Ratiopharm, 0.2 mg/mouse, subcutaneously), and chlorprothixene (Sigma, 0.2 mg/mouse, intramuscularly). Following injections, animals were placed in a stereotaxic frame. Animals’ body temperature was maintained at 37 °C and heart rate was monitored throughout the experiment. Inhalation anesthesia was maintained with 0.6–0.8 % halothane in a mixture of O_2_:N_2_O (1:1). Lidocaine (2 % xylocain jelly) was applied locally to all incisions. The skin above the skull was incised to expose V1 of the left hemisphere, and agarose (2.5 % in 0.9 % NaCl) and a glass cover-slip were placed over the exposed area. Mouse visual cortical responses were recorded through the intact skull using the imaging method developed by Kalatsky and Stryker (2003) and optimized for the assessment of OD plasticity by Cang et al. (2005). In this method, a temporally periodic stimulus is continuously presented to the animal and the cortical responses at the stimulus frequency are extracted by Fourier analysis. Optical images of intrinsic cortical signals were obtained using a CCD camera (Dalsa) controlled by custom software. The surface vascular pattern and intrinsic optical signal images were visualized with illumination wavelengths set by a green (550 ± 10 nm) or red (610 ± 10 nm) interference filter, respectively. After acquisition of a surface image, the camera was focused 600 μm below the cortical surface. An additional red filter was interposed between the brain and the CCD camera. Frames were acquired at a rate of 30 Hz, temporally binned to 7.5 Hz and stored as 512 × 512 pixel images after spatial binning of the camera image.

*Visual stimuli* Drifting horizontal bars (2° wide) were presented to the animal at a distance of 25 cm on a high refresh rate monitor. The distance between two bars was 70° and they were presented at a temporal frequency of 0.125 Hz. For calculating ocular dominance, the visual stimulus was restricted to the binocular visual field of the left V1 (−5° to +15° azimuth) and animals were stimulated through either the left or the right eye in alternation. For determining the quality of retinotopic maps, we used full-field stimulation through the contralateral eye with a horizontal (elevation maps) or vertical (azimuth map) moving bar, extending 62 × 92° of the visual field contralateral to the recorded hemisphere.

*Data analysis* Visual cortical maps were calculated from the acquired frames by Fourier analysis to extract the signal at the stimulation frequency using custom software (Kalatsky and Stryker 2003). While the phase component of the signal is used for the calculation of retinotopy, the amplitude component represents the intensity of neuronal activation (expressed as fractional change in reflectance ×10^−4^) and was used to calculate OD. For that, the ipsilateral eye magnitude map was first smoothed to reduce pixel shot noise by low-pass filtering using a uniform kernel of 5 × 5 pixels, and then thresholded at 30 % of peak response amplitude to eliminate the background noise. Then an OD index (ODI) for every pixel in this region was calculated as: (C − I)/(C + I), with C and I representing the response magnitudes of each pixel to visual stimulation of the contralateral and ipsilateral eye, respectively (Cang et al. 2005). The ODI ranges from −1 to +1, with negative values representing ipsilateral and positive values representing contralateral dominance. We then computed an ODI as the average of the OD scores of all responsive pixels. Consequently, we calculated ODIs from blocks of 4 runs in which the averaged maps for each eye had at least a response magnitude of 1 × 10^−4^. All ODIs of one animal (typically 3–5) were averaged for further quantification and data display. In the polar maps, hue encodes visual field position (retinotopy) and lightness encodes the magnitude of the visual responses. The quality of the retinotopic maps was assessed by the calculation described by Cang et al. (2005) on contralateral eye maps of 3–4 mice. We selected the most responsive area (in the number of 20,000 pixels) within V1 by thresholding at 30 % of peak response amplitude of the activity map. For each of the pixels within this area the difference between its visual field position and the mean position of its surrounding 24 pixels was calculated. For maps of high quality, the position differences are quite small because of smooth progression. The standard deviation of the position difference was then used as an index of the quality of retinotopic maps with small values indicating high map quality and vice versa. Additionally, the size of the V1 maps was calculated using MATLAB.

**Fig. 1:**
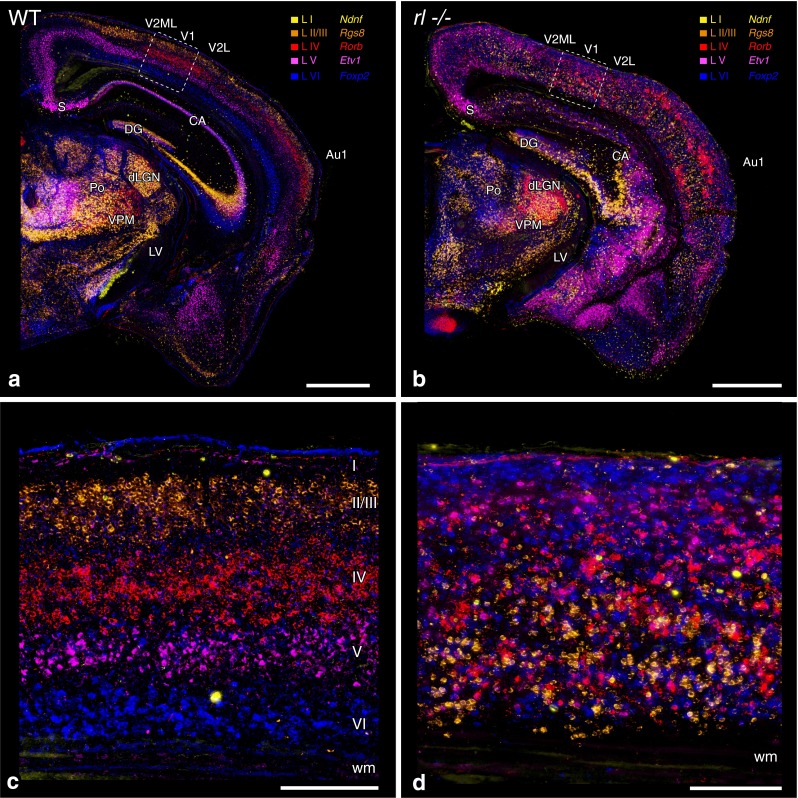
Lamina-specific markers reveal the highly disrupted lamination of the *reeler* visual cortex. **a**, **b** Low-magnification coronal view of the wild type (WT, **a**) and *reeler* (*rl*−/−, **b**) brain, illustrating the layer-specific mRNA expression in the WT cortex and the disorganized lamination in the *rl*−/− cortex. Note that primary sensory areas (V1 and Au1) can be identified from their pronounced *Rorb* expression (*red*). **c, d** Higher magnification of the V1 area delineated by the dashed frames in **a**, **b**. The WT cortex **c** shows the well-known (*vertical*) compartmentalization into cortical layers (*Roman numerals*), whereas the *rl*−/− cortex **d** does not show any obvious layering. All pictures were compiled from the merging of five pseudo-colored micrographs from consecutive sections (chromogenic in situ hybridizations). *Au1* primary auditory cortex, *CA* cornu ammonis, *DG* dentate gyrus, *dLGN* dorsal lateral geniculate nucleus, *LV* lateral ventricle, *Po* posterior thalamic nuclear group, *S* subiculum, *V1* primary visual cortex, *V2L* secondary visual cortex, lateral area, *V2ML* secondary visual cortex, mediolateral area, *VPM* ventral posteromedial thalamic nucleus, *wm* white matter. *Scale bars* **a**, **b** 1,000 µm, **c**, **d** 200 µm

**Fig. 2:**
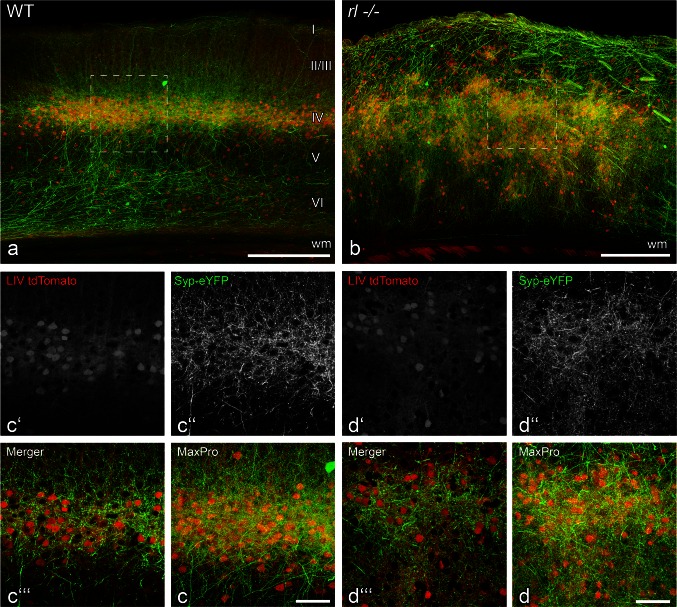
Thalamic fibers connect to their layer IV-fated target cells despite of the vertical disorganization of the *reeler* visual cortex. **a, b** Axonal projections from the dLGN visualized by eYFP coupled Synaptophysin (Syp-eYFP; green) into WT (**a**, **c**) and *rl*−/− (**b**, **d**) V1. Layer IV-fated target cells express tdTomato (Cre-dependent under the layer-specific Scnn promotor; *red*). **c d** Higher magnification of the V1-area delineated by the *dashed frames* in **a**, **b** (maximum intensity projection of 50 *z* planes). **c′ d′** Layer IV-fated cortical cells (single optical plane); **c″ d″** thalamic axons and their boutons (single optical plane); **c‴ d‴** Merger. Note the shape of thalamic terminal fields that follows the distribution of layer IV cells in the WT as well as the disorganized *rl*−/− brain. *Scale bars* **a**, **b** 250 µm; **c**, **d** 50 µm

*Acquisition of histological/immunofluorescent images* Brightfield images (Fig. 1) were taken with an Axio Imager M2 (Zeiss) controlled by a Neurolucida system (mbf Bioscience). The images were acquired as virtual tissues (VTs; 6 z planes, 5-µm steps) and are presented as minimum intensity projections. Micrographs of consecutive sections were assigned to a respective false color and merged using Photoshop CS6 (Adobe). Wide-field fluorescence VTs for data analysis were acquired using the same setup and acquisition procedure (i.e. VTs with 5 µm step size; Fig. 8a, b), but presented as maximum intensity projections. The images in Fig. 8c–f were acquired using a structured illumination technique (ApoTome; Zeiss). The resulting VTs consist of 30 z planes with 1 µm step size and are presented as maximum intensity projections as well. Confocal images (Fig. 2) were acquired using a spectral confocal (Leica TCS SP2). The Leica confocal software was controlled by the Vision4d software (Arivis) to acquire and stitch multiple, predefined tiles.

### Data and statistical analyses

All intra- and intergroup comparisons were analyzed either by a two-tailed *t* test or one-way ANOVA. The intergroup comparison of the enhancement of visual acuity, and contrast sensitivity as well as VWT performance was analyzed by two-way ANOVA with repeated measurements and Bonferroni correction. Maximum intensity projections of the PV/WFA staining were analyzed by manual counting using the Neurolucida software. The number of PV^+^ cells and PNNs was obtained from consecutive micrographs of V1 (in 40-µm-thick sections). Data were exported from the Neurolucida-Explorer (mbf Bioscience) to Excel (Microsoft). Statistical analysis was done using Sigma Plot 12 (Systat). Significance was tested using two-tailed t tests. The level of significance was set to *p < 0.05; **p < 0.01; ***p < 0.001. Data are represented as mean ± SEM (if not stated differently).

## Results

### Neurons with different laminar fates are intermingled in *rl*−/− V1

While the structural organization of the neocortex in the absence of reelin has been extensively studied (D’Arcangelo 2005), a new methodological approach based on laminar-specific RNA probes (Lein et al. 2007) has demonstrated that the extent of cortical disorganization in the somatosensory cortex was previously underestimated (Wagener et al. 2010). Therefore, a fine-grained structural analysis of *rl*−/− V1 was the first step in our analysis. We used RNA probes specific for the following layers: *Ndnf* for layer I, *Rgs8* for layer II/III, *Rorb* for layer IV, *Etv1* for layer V, and *Foxp2* for layer VI (Lein et al. 2007; Boyle et al. 2011). In the neocortex of the WT brain, labeling revealed the well-known compartmentalization into the six neocortical layers (Fig. 1a). A pronounced expression of the layer IV marker (*Rorb*) allowed for the unambiguous identification of V1. Hybridization of the probes in the *rl*−/− brain revealed massive structural disorganization of the neocortex (Fig. 1b). The different populations of marker-labeled cells were extensively intermingled. However, similar to the WT neocortex, dense expression of the layer IV marker (*Rorb*) allowed for the delineation of V1 in its typical position.

A higher magnification confirmed the presence of a well-ordered laminar organization in the WT brain (Fig. 1c). By contrast, a closer look at *rl*−/− V1 (Fig. 1d) did not demonstrate an obvious structural organization and showed a complex distribution of the different marker-labeled cellular populations. *Foxp2*-expressing cells, which normally are present close to the white matter, were concentrated in but not restricted to an area close to the pial surface, whereas *Rgs8*-expressing cells, which can normally be found in the upper cortical layers, were preferentially located in but again not restricted to the lower half of the visual cortex. Thus, simply describing the pattern as an inversion fails to capture the true scope of the disorganization. In general, both *Foxp2*- and *Rgs8*-expressing cells were distributed over the whole cortical thickness. Roughly the same held true for *Rorb*- and *Etv1*-expressing cells, the difference being that these two populations were absent in a restricted area below the pial surface and above the white matter. The density of *Etv1* cells peaked below the field of highest *Foxp2* density. The few *Ndnf*-expressing cells appeared to be randomly distributed. Altogether, the pattern of neurons with different laminar fates in *rl*−/− V1 has to be regarded as highly disrupted with only a subtle tendency of an inversion. Thus, the magnitude of disorganization strongly exceeds previous descriptions that focused on an inversion and proposed fuzzy borders of the laminar compartments (Caviness 1982; D’Arcangelo 2005).

### Visual information reaches the disorganized *rl−/−* cortex via correctly targeted projections from the dorsal lateral geniculate nucleus (dLGN)

In the visual system, afferent sensory input from the dLGN is transmitted by a precise projection mainly to cortical layer IV cells (Simmons et al. 1982; Antonini et al. 1999). Based on our laminar fate-marker results, we asked whether these fibers are still able to target layer IV-fated cells, i.e. their preferential input compartment, in the disorganized *rl*−/− cortex. Therefore, we injected a viral vector expressing eYFP-tagged presynaptic synaptophysin (Syp) into the dLGN and visualized the postsynaptic layer IV-fated cells using a transgenic “layer IV tdTomato animal” (see “Materials and methods”). Stereotactic injections into the WT brain revealed a substantial overlap of Syp-eYFP-expressing thalamic boutons and the tdTomato-expressing layer IV cells (Fig. 2a, c–c‴). This overlap strongly suggests that thalamic fibers form contacts on layer IV cells. In agreement with the in situ hybridization analysis for the layer IV marker *Rorb*, the transgenic layer IV-fated cells in the *rl*−/− cortex were distributed over extensive parts of the cortical thickness, forming small but discrete tdTomato-labeled cell clusters (Fig. 2b). To a large extent, these clusters also overlapped with the eYFP-labeled thalamic boutons, suggesting that the thalamic fibers preferentially target layer IV-fated cells, no matter where in the cortex these cells end their migration (Fig. 2b, d–d‴). Thus, despite the ectopic position of layer IV-fated cells in the *rl*−/− neocortex, thalamocortical information transmission is probably based on the same recipient cells as in the WT brain.

### The basic functional organization of visual cortex and visual performance are largely normal in reelin-deficient mice

**Fig. 3:**
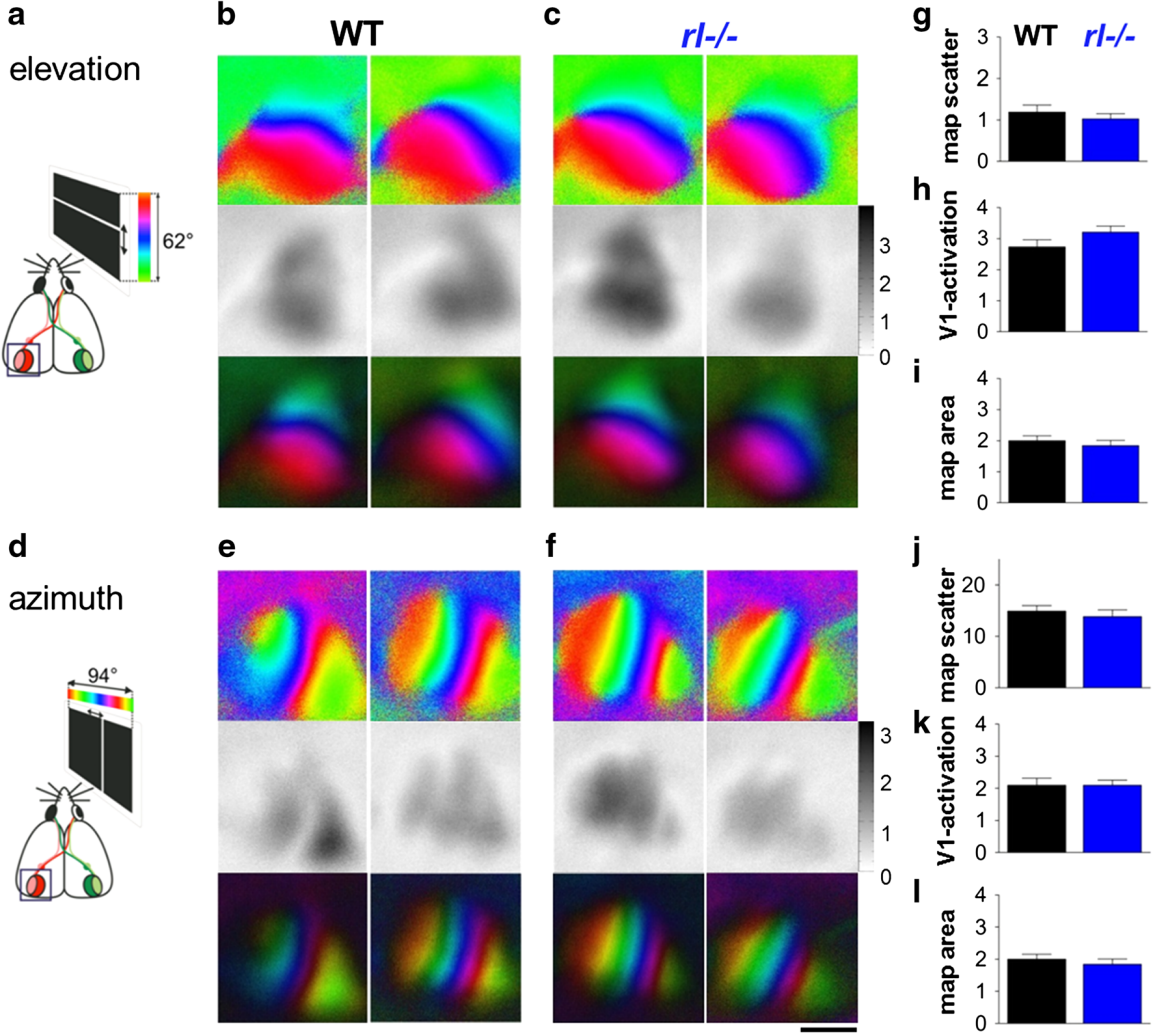
The basic organization of visual cortical maps of *rl*−*/*− mice is indistinguishable from WT mice. Representative examples of retinotopic and activity maps visualized with intrinsic signal optical imaging in primary visual cortex (V1) of both genotypes. Color-coded phase maps (*top*), grey-scale coded response magnitude maps (*middle*) and polar maps of retinotopy (*bottom*), and their quantification (g^–l^) are illustrated. The magnitude of the optical responses is displayed as fractional change in reflection ×10^−4^. Retinotopic maps are color-coded according to the schemes on the *left* side (**a**, **d**). Both elevation (**b**, **c**) and azimuth (**e**, **f**) maps resulting from visual stimulation of the animals with moving *horizontal* (**a**) or *vertical* *bars* (d) are illustrated. Quantifications of V1 activation (**h**, **k**), retinotopic map quality (map scatter, **g**, **j**) and map area in mm^2^ (**i**, **l**). Mean ± SEM, for all *p* > 0.05

*V1*-*maps* Despite the highly disorganized layering in *rl*−/− V1, optical imaging of intrinsic signals revealed retinotopic maps and a magnitude of sensory-driven activation that were basically indistinguishable from WT littermates. Both elevation maps induced by visual stimulation with horizontal moving bars (Fig. 3a–c) and azimuth maps induced by visual stimulation with vertical moving bars (Fig. 3d–f) were qualitatively and quantitatively similar to WT maps, suggesting a normal topographic activation. There were no significant differences between WT and *rl*−/− mice in map quality, V1-activation and map size (WT/*rl*−/− (*n* = 8/7), elevation maps (Fig. 3g–i): map scatter: 1.2 ± 0.2/1.0 ± 0.1; V1 activation: 2.7 ± 0.2/3.2 ± 0.2; map area: 2.8 ± 0.1 mm/2.4 ± 0.2 mm^2^; azimuth maps (Fig. 3j–k): map scatter: 14.9 ± 1.1/13.9 ± 1.3, V1 activation: 2.1 ± 0.2/2.1 ± 0.2; map area: 2.0 ± 0.2/1.8 ± 0.2 mm^2^, *t* test, *p* > 0.05 for all comparisons). These data confirm and extend previous findings (Drager 1981; Simmons and Pearlman 1982), suggesting that the abnormality in neuronal positioning in *rl*−/− mice does not interfere with the formation of retinotopically ordered afferent connections or affect the magnitude of visual cortical activation or the size of the activated area.

### Basic visual performance is similar in *rl−/−* and WT mice

**Fig. 4:**
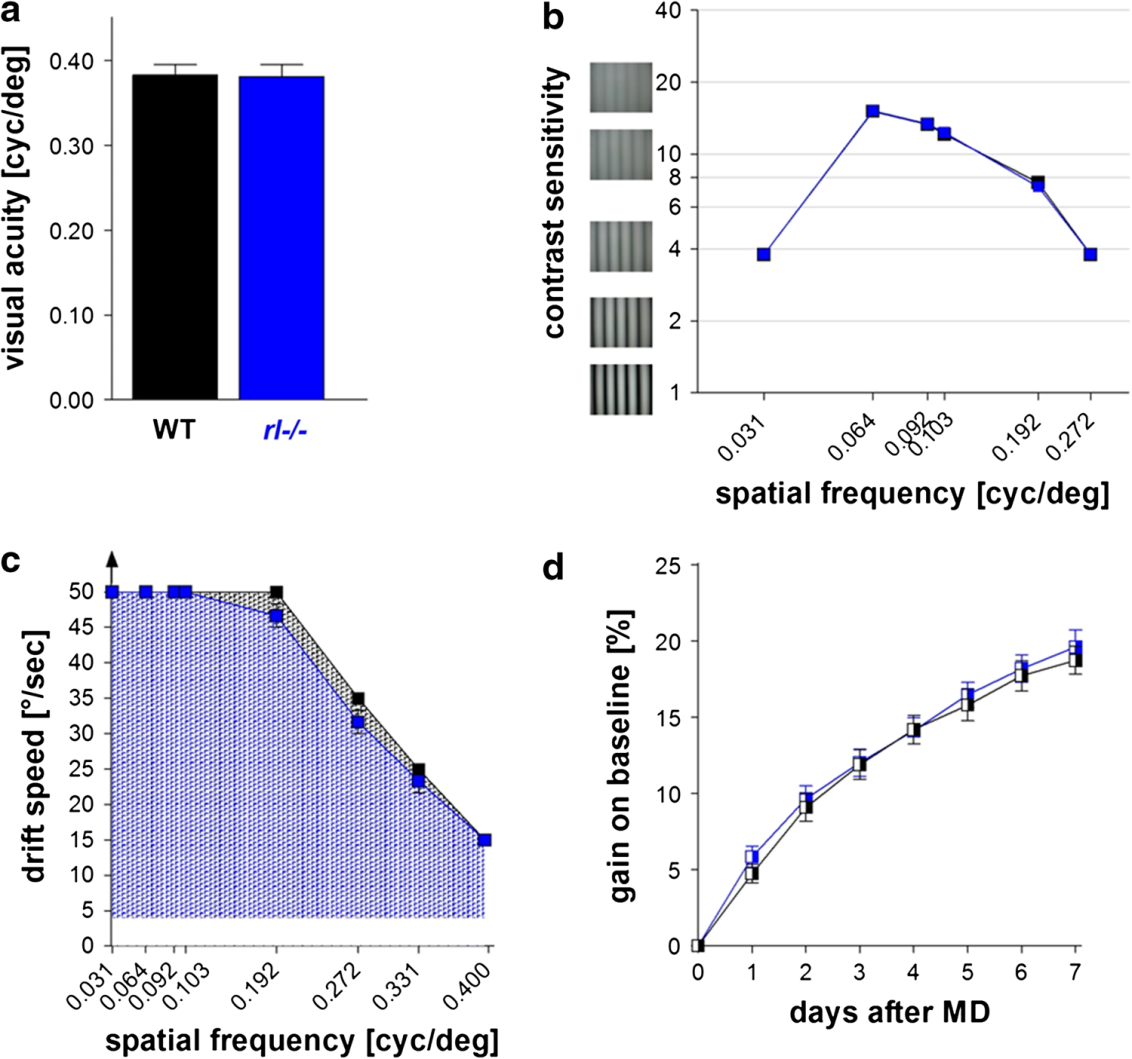
Basic visual performance is similar in WT and *rl*−/− mice. Visual acuity (**a**), contrast sensitivity (**b**) and temporal frequency thresholds (**c**) of the optomotor reflex measured with the virtual optomotor system in WT (*black*, *n* = 14) and *rl*−/− (blue, *n* = 16) mice. Contrast sensitivity (**b**) and drift speed (**c**) plotted as a function of the spatial frequency of the visual stimuli. **d** Gain on baseline (in %) of the visual acuity improvements over 7 days of monocular deprivation (MD) and daily testing. Mean ± SEM, for all *p* > 0.05

Since the formation of ordered retinotopic maps does not necessarily indicate unimpaired V1-function, we additionally measured visual performance of *rl*−/− and WT littermates. It was shown that the retina of *rl*−/− mice displayed an abnormal stratification pattern of synaptic connections between rod bipolar cells and amacrine cells and the number of rod bipolar cells was reduced (Rice et al. 2001). Nevertheless, it was not clear if such retinal abnormalities affect visual abilities of *rl*−/− mice. Using the virtual-reality optomotor setup (Prusky et al. 2004), we measured visual acuity, contrast sensitivity and temporal frequency thresholds of the optomotor reflex in both *rl*−/− and WT mice (Fig. 4). Essentially, there were no significant differences in all measured parameters (*p* > 0.05 for all comparisons). The visual acuity threshold of the optomotor reflex was on average 0.38 ± 0.004 cycles/degree (cyc/deg) in WT (*n* = 14) and 0.40 ± 0.03 cyc/deg in *rl*−/− (*n* = 16) (Fig. 4a). Likewise, values for contrast sensitivity and temporal resolution were indistinguishable between WT and *rl*−/− mice (Table 1; Fig. 4b, c). Our present data thus show that reelin deficiency neither affects the optomotor reflex nor basic visual capabilities, confirming and extending previous observations that grating sensitivity was not different between WT and *rl*−/− mice (Sinex et al. 1979).

**Table 1:** Optomotry-measured contrast sensitivity and temporal resolution

Spatial frequency (cyc/°)	Contrast sensitivity		Temporal resolution (°/sec)	
	WT	*rl*−/−	WT	*rl*−/−
0.031	3.8 ± 0.03	3.8 ± 0.02	50.0 ± 0.00	50.0 ± 0.00
0.064	15.1 ± 0.39	15.2 ± 0.24	50.0 ± 0.00	50.0 ± 0.00
0.092	13.3 ± 0.35	13.4 ± 0.25	50.0 ± 0.00	50.0 ± 0.00
0.103	12.1 ± 0.27	12.3 ± 0.25	50.0 ± 0.00	50.0 ± 0.00
0.192	7.6 ± 0.19	7.3 ± 0.18	50.0 ± 0.00	46.7 ± 0.17
0.272	3.8 ± 0.04	3.8 ± 0.04	35.0 ± 0.00	31.7 ± 0.17
0.331			25.0 ± 0.00	23.3 ± 0.17
0.400			15.0 ± 0.00	15.0 ± 0.00

### Experience-dependent enhancement of the optokinetic response is preserved in *rl−/−* mice

Since the optomotor reflex is mediated by subcortical circuitry and therefore might not directly be influenced by the disorganized cortical lamination of *rl*−/− mice, we next tested whether the experience-dependent enhanced vision of the non-deprived eye that depends on the visual cortex (Prusky et al. 2006) is compromised in *rl*−/− mice. To this end, we measured the spatial frequency threshold of the optomotor reflex in both genotypes before and after monocular deprivation (MD). Both groups of mice exhibited the typical enhancement of the spatial frequency sensitivity of the optokinetic response of the open eye during the 7 days of MD and daily testing. In WT mice, visual acuity of the optomotor reflex increased from 0.38 ± 0.001 cyc/deg on day 0 to 0.45 ± 0.004 cyc/deg on day 7 after MD (*n* = 8; *t* test, *p* < 0.001). In *rl*−/− mice, values increased from 0.37 ± 0.002 cyc/deg to 0.45 ± 0.003 on day 7 after MD (*n* = 10; *t* test, *p* < 0.001). On average, values increased by 19 ± 1 % in WT and by 20 ± 1 % in *rl*−/− mice (Fig. 4d). Contrast sensitivity also increased significantly in both genotypes (two-way ANOVA, *p* < 0.001, Table 2). Taken together, there was no difference between the genotypes for both sets of data (two-way ANOVA, *p* > 0.05), showing that reelin deficiency did not compromise the experience-dependent enhancement of vision after MD.

**Table 2:** Optomotry-measured contrast sensitivity improvements after MD

Spatial frequency (cyc/°)	Contrast sensitivity			
	Day 0—noMD		Day 7—MD	
	WT	*rl*−/−	WT	*rl*−/−
0.031	3.4 ± 0.4	3.7 ± 0.02	5.1 ± 0.09	4.8 ± 0.05
0.064	15.8 ± 0.2	15.0 ± 0.24	27.2 ± 1.30	25.2 ± 0.66
0.092	13.6 ± 0.14	13.1 ± 0.19	24.5 ± 1.39	21.1 ± 0.73
0.103	12.6 ± 0.17	12.3 ± 0.16	21.6 ± 1.30	19.1 ± 0.59
0.192	7.5 ± 0.15	6.9 ± 0.17	12.4 ± 0.38	11.3 ± 0.52
0.272	3.7 ± 0.02	3.7 ± 0.03	4.9 ± 0.10	4.7 ± 0.03

### Reelin-deficient mice show reduced orientation discrimination, but learn and recall the visual discrimination task

**Fig. 5:**
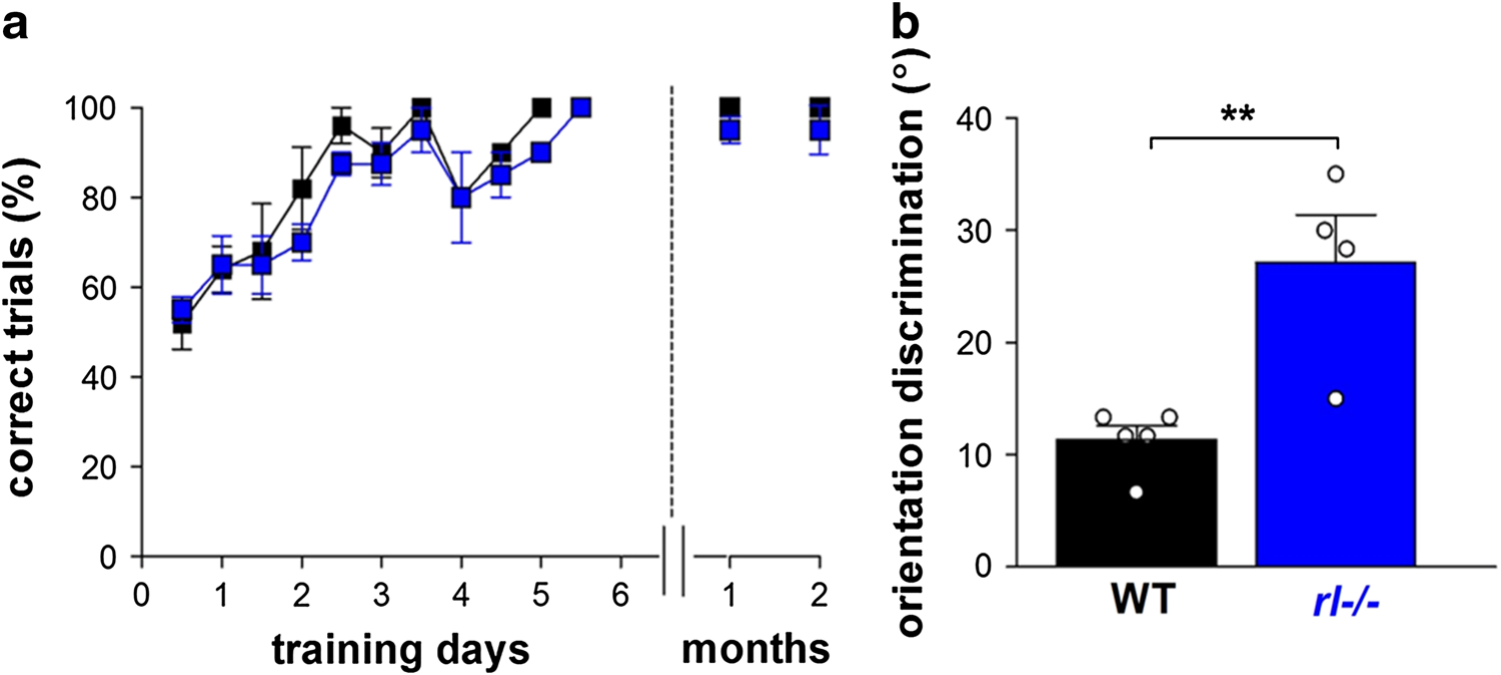
*Rl*−/− mice show reduced orientation discrimination but intact learning and recall in the visual water task. **a** Learning curve for the orientation discrimination task in the VWT for WT (*n* = 5, *black*) and *rl*−/− (*n* = 4, *blue*) mice, displayed as correct trials vs. training days for the initial learning phase (*left*) and recall 1 and 2 months later (*right*). **b** Orientation discrimination in degree (°) of WT and *rl*−/−mice. *Circles* illustrate values of individual animals; Mean ± SEM, **p < 0.01

Since *rl*−/− mice were neither compromised in basic visual abilities nor in the experience-driven enhancement of vision after MD, we next tested how they perform in a more challenging perceptual task: orientation discrimination. Initially, WT and *rl*−/− mice had to learn to swim towards the rewarded grating below which an escape platform was located. There was no difference in the learning curve between the genotypes: WT and *rl*−/− mice reached the criterion (3 × 90 % correct) within on average 3.5 ± 0.4 and 4.2 ± 0.5 days (WT/*rl*−/−, *n* = 5/4, two-way ANOVA, *p* > 0.05; Fig. 5a). Then, the discrimination threshold of individual mice was determined by gradually decreasing the orientation difference of the rewarded and distractor grating until performance fell below 70 % accuracy. Orientation discrimination was impaired in *rl*−/− mice compared to WT littermates: while WT mice perceived a minimal orientation difference of 11 ± 3˚, *rl*−/− mice required 27 ± 3˚ and thus a much larger orientation contrast for a correct behavioral decision (*t* test, *p* < 0.01, Fig. 5b). Thus *rl*−/− animals learned the task as WT, but performed worse and showed reduced orientation discrimination ability. In addition, after 1 and 2 months, mice of both genotypes remembered the task similarly since there was no difference in the percentage of correct trials (Fig. 5a, right panel, two-way ANOVA, *p* > 0.05). Together, these results show that reelin deficiency and the accompanying structural malformations in both cortical and hippocampal circuits do not affect the ability to learn and remember a visual discrimination task, but rather affect more elaborate visual performance.

### *rl−/−* mice preserve a juvenile form of ocular dominance (OD) plasticity into late adulthood

**Fig. 6:**
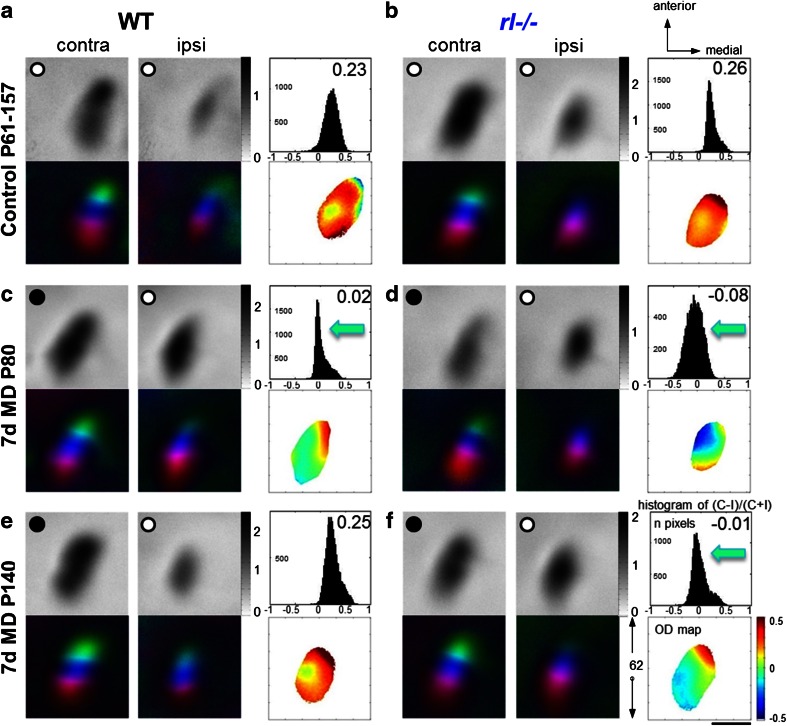
*Rl*−/− mice preserve ocular dominance plasticity after MD into late adulthood. Representative examples of optically recorded activity maps after visual stimulation of the contra- (contra) and ipsilateral (ipsi) eye in the binocular region of mouse V1 in adult WT (**a**, **c**, **e**) and *rl*−/− mice (**b**, **d**, **f**). Maps of WT and *rl*−/− mice without MD are shown in the *top row* (control), maps after 7 days of MD in P80 mice in the *middle* (**c**, **d**), and maps of P140 mice at the bottom (**e**, **f**). *Grey-scale* coded response magnitude maps and their quantification (*top*), and color-coded polar maps of retinotopy (*bottom*) are shown. In both genotypes without MD, activity patches evoked by stimulation of the contralateral eye were darker than those after ipsilateral eye stimulation, the average ODI was positive, and warm colors prevailed in the 2-dimensional OD map, indicating contralateral dominance (**a**, **c**). In P80 mice, 7 days of MD induced a significant OD shift towards the open eye in both genotypes. In contrast, in P140 mice, OD plasticity was present only in *rl*−/− mice. *Scale bar* 1 mm

**Fig. 7:**
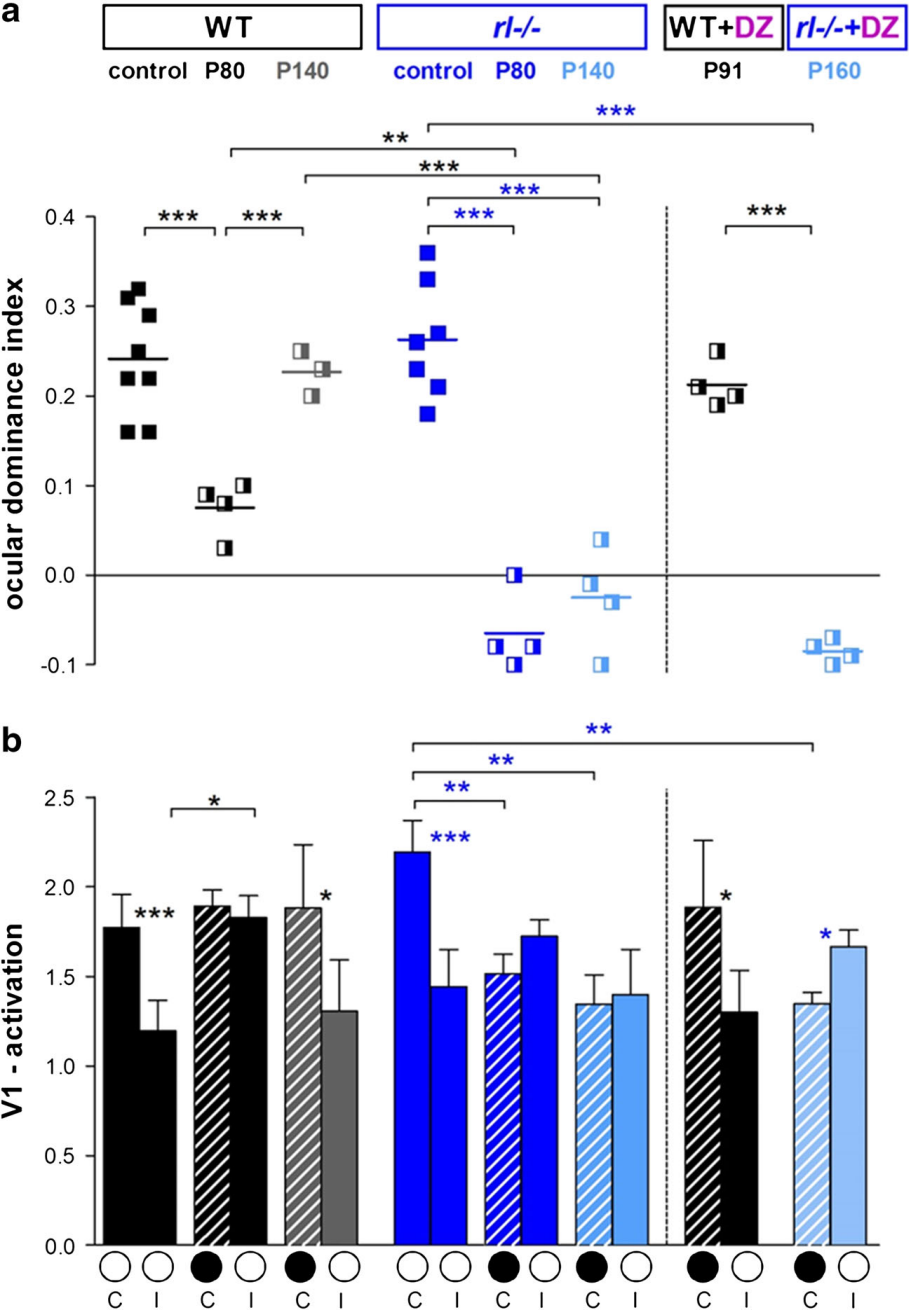
*Rl* −/− mice show juvenile-like OD plasticity into late adulthood **a** Optically imaged OD indices in control animals (*filled squares*) and after 7 days of MD (*half-filled squares*). Values from WT mice are shown in *black* (control, P80) and grey (P140), those of *rl*−/− mice in blue (control, P80) and *light blue* (P140). *Symbols* illustrate ODI values of individual animals; means are marked by *horizontal lines*. **b** V1-activation elicited by stimulation of the contralateral (C) or ipsilateral (I) eye in control animals and after MD (*black filled circle* indicates MD eye) of all experimental groups shown in a (same color code as in **a**). In WT P80 mice, OD shifts were mediated by an increase in open (I) eye responses. In contrast, *rl*−/− mice showed not only significantly stronger OD shifts at P80, but also continued to exhibit OD plasticity at P140. Interestingly, OD shifts of *rl*−/− mice were always mediated by a reduction of deprived (C) eye responses in V1. Diazepam treatment (+DZ) completely abolished OD shifts after MD in WT (WT + DZ) but not in *rl*−/− mice (*rl*−/− +DZ). Mean ± SEM, *p < 0.05;**p < 0.01; ***p < 0.001

To challenge the disorganized visual cortical circuits, we compared the effects of MD on OD plasticity in V1 of both *rl*−/− mice and their WT littermates. While it has been shown previously that cells in the binocular region of *rl*−/− V1 receive convergent input from the two eyes and that projections from the retina are stronger onto the contralateral hemisphere (Drager 1981), nothing is known about how the disturbed lamination or lack of reelin might influence experience-dependent changes of visual cortical networks. For imaging V1 plasticity, visual stimuli were restricted to the binocular part of the visual field of the recorded hemisphere and V1 activity elicited by both ipsilateral and contralateral eye stimulation was recorded optically. In both genotypes, V1 activity induced by stimulation of the contralateral eye was higher than that induced by ipsilateral eye stimulation (Fig. 6a, b). Thus, the OD index (ODI) was positive, indicating contralateral eye dominance. After 7 days of MD in adult (postnatal day (P) 80) *rl*−/− and WT mice, both eyes activated V1 more equally strong and the ODI was reduced, such that there was an OD shift towards the open eye (Fig. 6c, d). Interestingly, the OD shift of the *rl*−/− mice was stronger than that of the WT mice: the average ODI of WT mice decreased from 0.23 ± 0.02 (*n* = 8) to 0.07 ± 0.01 after 7 days of MD (*n* = 4), while in *rl*−/− mice it decreased from 0.26 ± 0.02 (*n* = 7) to −0.06 ± 0.02 after MD (*n* = 4, for both ANOVA, *p* < 0.001, Fig. 7a). Thus, the size of the OD shift of *rl*−/− mice after 7 days of MD basically resembled OD shifts in 4-week-old WT mice after 4 days of MD (Lehmann and Löwel 2008; Sato and Stryker 2008). In addition, quantitative analysis of ODIs and V1 activations revealed that OD shifts of *rl*−/− mice were mediated by a different mechanism (Fig. 7b): while in P80 WT mice—as shown for 3-month-old C57Bl6J mice (Lehmann and Löwel 2008; Sato and Stryker 2008)—OD shifts were mediated by an increase of open eye responses in V1, but in *rl*−/− mice, OD shifts were mediated by a reduction of deprived eye responses, a typical sign of juvenile OD plasticity. Without MD, OD indices and V1 activation through the contra- and ipsilateral eye were not different between the genotypes (V1 activation: ipsi, WT, 1.2 ± 0.02, vs. *rl*−/−, 1.4 ± 0.2; contra, WT, 1.8 ± 0.2 vs. *rl*−/−, 2.2 ± 0.2; ANOVA, *p* > 0.05). After MD in P80 WT mice, there was no change in V1 activation through the deprived eye (no MD 1.8 ± 0.18 vs. MD 1.9 ± 0.1, *t* test, *p* > 0.05), but open eye activity increased (no MD 1.2 ± 0.16 vs. MD 1.8 ± 0.12, *t* test, *p* < 0.01, Fig. 7b). In contrast, in P80 *rl*−/− mice, V1 activity driven by the deprived eye decreased from 2.2 ± 0.2 to 1.5 ± 0.1 after MD (*t* test; *p* < 0.05), while ipsilateral eye activities did not change (no MD: 1.4 ± 0.2, with MD: 1.7 ± 0.1; *t* test, *p* > 0.05, Fig. 7b). Additionally, after MD, the ODI of P80 *rl*−/− mice was significantly lower compared to age-matched WT mice (WT, 0.07 ± 0.01 vs. -0.06 ± 0.02, *t* test, *p* < 0.01).

*OD* *plasticity in P140 rl*−/− *mice.* The enhanced plasticity in the P80 *rl*−/− mice prompted us to also investigate OD plasticity in even older animals. Surprisingly, OD shifts towards the open eye were still present in P140 *rl*−/− mice after MD, while age-matched WT mice did no longer display OD plasticity, as expected given our previous data (Lehmann and Löwel 2008). In WT mice, the deprived eye continued to dominate V1 (Fig. 6e), while in *rl*−/− mice, both eyes activated V1 equally strong (Figs. 6f, 7b). After 7 days of MD, the ODI of P140 *rl*−/− mice was −0.03 ± 0.03 (*n* = 4, ANOVA, *p* < 0.001), and the OD shift was mediated by a significant reduction of deprived eye responses in V1 (no MD, 2.2 ± 0.17 vs. MD, 1.3 ± 0.16, ANOVA, *p* < 0.05) while open eye responses did not change (no MD, 1.4 ± 0.2 vs. MD, 1.4 ± 0.25, ANOVA, *p* > 0.05). In contrast, P140 WT mice neither showed an OD shift after MD (no MD *n* = 7, 0.23 ± 0.02 vs. MD, *n* = 3, 0.22 ± 0.01, ANOVA, *p* > 0.05) (Fig. 7) nor displayed any changes in visually driven activity in V1 through the deprived or open eye (deprived eye: no MD 1.8 ± 0.2 vs. MD 1.9 ± 0.4, *p* > 0.05; open eye: no MD, 1.2 ± 0.2 vs. MD, 1.3 ± 0.3, ANOVA, *p* > 0.05). Taken together, *rl*−/− mice retained a juvenile-like OD plasticity into late adulthood, suggesting that reelin deficiency may cause some structural and/or functional changes in neuronal circuitry that promote cortical plasticity.

### Diazepam has no effect on OD plasticity in *rl−/−* mice

Since developmental strengthening of local inhibitory circuits contributes to the termination of the critical period for OD plasticity (Hensch et al. 1998; Fagiolini et al. 2004; Sugiyama et al. 2008; Jiang et al. 2010) and experimental reduction of the inhibitory tone can partially restore OD plasticity in older rodents (Maya Vetencourt et al. 2008a; Harauzov et al. 2010; Morishita et al. 2010; Baroncelli et al. 2011), it is possible that the preserved juvenile OD plasticity in adult *rl*−/− mice was the result of a reduction in inhibitory tone. We tested this by in vivo pharmacological manipulations with diazepam. Diazepam has been used previously to increase the GABAergic tone in pre-critical period mice to trigger the onset of the critical period for OD plasticity and to block restored plasticity after reduction of inhibition in older rodents (Hensch et al. 1998; Fagiolini et al. 2004; Maya Vetencourt et al. 2008a; Morishita et al. 2010; Greifzu et al. 2014). While diazepam treatment during the 7 days of MD completely and reliably blocked OD shift in adult WT mice, confirming the important role of the inhibitory system for cortical plasticity, diazepam did not abolish OD plasticity in adult *rl*−/− mice (Fig. 7). After 7 days of MD, diazepam-treated WT mice had an ODI of 0.21 ± 0.01 (*n* = 4), not different from values of mice without MD (ANOVA, *p* > 0.05, Fig. 7a) but higher than in age-matched untreated mice with MD (*t* test, *p* < 0.001). In contrast, in *rl*−/− mice, diazepam did not block OD plasticity and the ODI was −0.08 ± 0.01 (*n* = 4), similar as in untreated *rl*−/− mice after MD (*t* test, *p* > 0.05), and reduced compared to *rl*−/− mice without MD (ANOVA, *p* < 0.001) or to diazepam-treated WT mice with MD (*t* test, *p* < 0.001) (Fig. 7a). Despite the diazepam treatment, the MD-induced OD shift in the P160 *rl*−/− mice remained exceedingly strong so that V1 even became dominated by visual stimulation of the previously weaker, ipsilateral eye (*t* test, *p* < 0.05, Fig. 7b). As without treatment, the preserved OD plasticity of *rl*−/−mice was mediated by a reduction of deprived eye responses after MD (1.3 ± 0.1, ANOVA, *p* < 0.001) while open eye responses did not change compared to *rl*−/− mice without MD (1.7 ± 0.1, ANOVA, *p* > 0.05). Thus, the preservation of juvenile OD plasticity in adult *rl*−/− mice is not likely mediated by changes in inhibitory tone. This conclusion is supported by our immunofluorescence data showing comparable numbers of PV^+^ cells in *rl*−/− and WT mice (see below).

### The number of PV^+^ cells and perineuronal nets (PNNs) is similar in adult *rl−/−* and WT mice

Maturation of inhibitory circuits is hypothesized to play an important role in termination of OD plasticity (Hensch 2005). Moreover, chondroitin sulfate proteoglycans (CSPGs), a component of the extracellular matrix, stabilize adult synaptic contacts and have been shown to be important molecular factors regulating visual cortical plasticity (de Vivo et al. 2013). Enzymatic degradation of CSPGs, which arrange into PNNs at the end of the critical period, restored OD plasticity in adult rats (Pizzorusso et al. 2002). Since PNNs preferentially enwrap PV^+^ inhibitory interneurons, their formation may limit structural plasticity by establishing a physical barrier that creates a non-permissive state for plasticity (Hartig et al. 1994; Ye and Miao 2013). Based on our finding of prolonged experience-dependent plasticity in the adult *rl*−/− brain we determined the density of both PV^+^ cells and PNNs in V1 of WT and *rl*−/− animals.

**Fig. 8:**
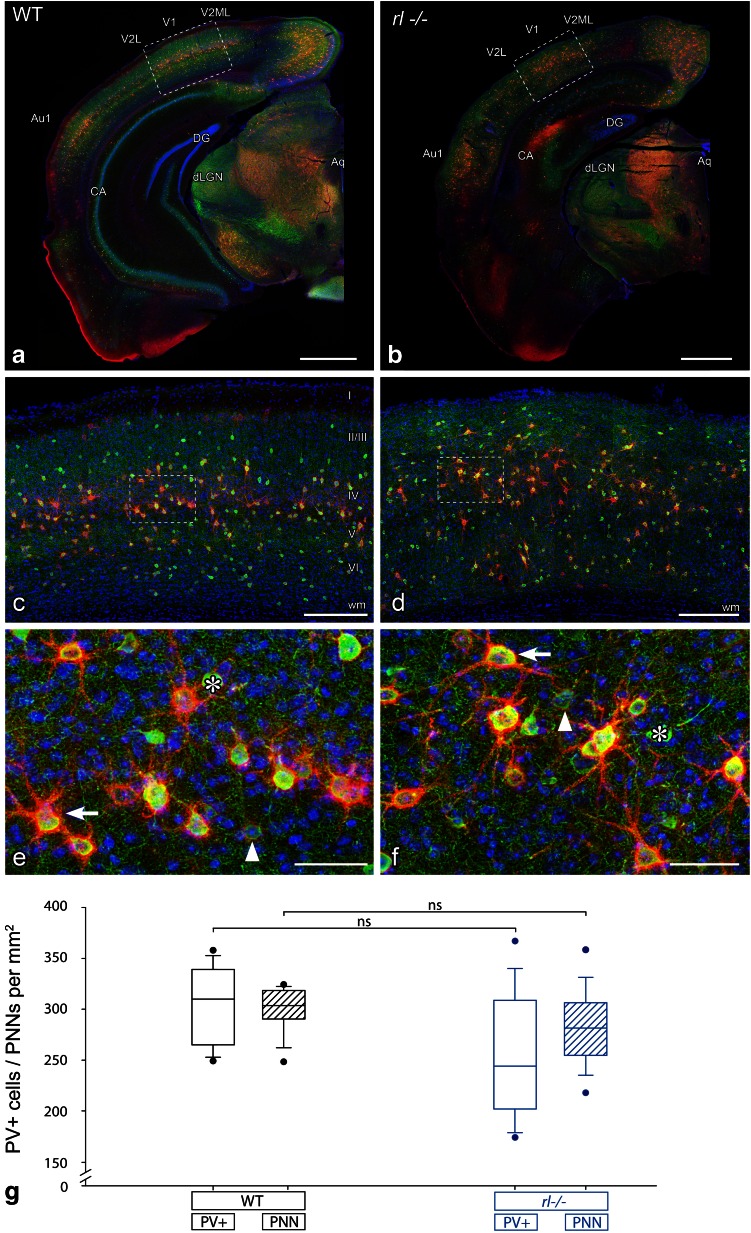
Similar numbers of parvalbumin positive (PV^+^) cells and perineuronal nets (PNNs) in V1 of wild type (WT) and *reeler* *(rl*−/−*)* mice. **a, b** Low-magnification image of the distribution of PV^+^ cells (*green*) and PNNs (labeled with Wisteria floribunda agglutinin; *red*) in the cortex, hippocampus, thalamus and midbrain of the WT (**a**) and *rl*−/−brain (**b**), sectioned in the standard coronal plane (*blue*, DAPI). Note that the primary visual and primary auditory cortex (V1 and Au1) can be identified from their higher density of PV^+^ cells and PNNs. **c, d** Higher magnification of the V1-area delineated by the *dashed frames* in **a**, **b**. PNNs in the WT cortex (**c**) show a strong preference for layers IV and V. In the *rl*−/− cortex (**d**), PNNs show a much broader distribution, in accordance with the disorganized layering (see Fig. 1). **e, f** Individual PV^+^ cells and PNNs (localization framed in **c**, **d**) in the WT (**e**) and *rl*−/− (**f**) cortex. Note that in both genotypes, PV^+^ cells with strong (*arrows*), weak (*arrowheads*), as well as absent (*asterisks*) PNNs can be found. **g** Quantification of the number of PV^+^ cells and PNNs presented as box plots. *Bottom* and *top* of the *boxes* indicate the 25th and 75th percentiles, respectively; the *bar* in the *box* is the median. The whiskers (*error bars*) indicate the 10th and 90th percentiles. Outliers are shown as *dots*. Neither the density of PV^+^ cells nor of PNNs showed significant differences between the genotypes. Abbreviations: *Aq* aqueduct (Sylvius), *Au1* primary auditory cortex, *CA* cornu ammonis, *DG* dentate gyrus, *dLGN* dorsal lateral geniculate nucleus, *V1* primary visual cortex, *V2L* secondary visual cortex, lateral area, *V2ML* secondary visual cortex, mediolateral area, *wm* white matter. *Scale bars* **a**, **b** 1,000 µm; **c**, **d** 250 µm; **e**, **f** 50 µm

V1 showed a high density of PV^+^ cells and PNNs, which allowed its easy delineation. This holds true both for the WT and disorganized *rl*−/− cortex (Fig. 8a, b). A closer view into the WT-V1 revealed a preferential location of PNNs in layer IV, where they frequently colocalized with PV^+^-interneurons (Fig. 8c). The PV^+^ cells showed strong, weak or absent PNNs (Fig. 8e). In the *rl*−/− V1, PNNs and PV^+^ cells seemed to be randomly distributed over nearly the whole cortical thickness (Fig. 8d), which is in agreement with the pronounced vertical misplacement of neurons (see Fig. 1). Nevertheless, PNNs showed the same general structure as in WT animals (Fig. 8f) and surrounded PV^+^-interneurons with strong or weak nets. In both *rl*−/− and WT animals, a substantial number of PV^+^ cells were not ensheathed by PNNs and some PNNs surrounded non-PV^+^ cells.

Quantification of the density of PNNs showed no significant differences between WT (*n* = 9) and *rl*−/− (*n* = 12) V1 (*t* test, *p* = 0.234; Fig. 8g). The same holds true for the density of PV^+^ cells (*t* test, *p* = 0.058; Fig. 8g). Thus, the strongly preserved experience-dependent plasticity in adult *rl*−/− V1 is not likely mediated by a reduced or loosened extracellular matrix, nor by a defective or absent maturation of PV^+^-circuits.

## Discussion

Using a combination of novel anatomical techniques, intrinsic signal optical imaging and behavioral tests, we investigated how the lack of the glycoprotein reelin influences the structure, function and plasticity of visual cortical circuits in adult mice. Our results revealed that lack of functional reelin does not simply invert, but highly disorganize visual cortical layers, resulting in a massive intermingling of cells with different laminar fates. This finding is in line with previous tracing approaches that already found a more severe phenotype of cortical disorganization based on radially scattered layer V corticotectal neurons in V1 (Baba et al. 2007; Yoshihara et al. 2010). Despite of this severe disorganization, afferent fibers from the dLGN preferentially innervate the layer IV-fated neurons, i.e. their correct target cells, irrespective of their ectopic positioning. *Rl*−/− mice also maintain both, normal basic visual capabilities and visual cortical maps with retinotopic organization and a similar stimulus-driven activation as WT mice, despite their cortical and reported retinal (Rice et al. 2001) abnormalities. In a challenging perceptual task, orientation discrimination, *rl*−/− mice were, however, compromised, but they learned and memorized this task as well as their WT littermates. Most strikingly, the lack of reelin not only enhanced but also prolonged experience-dependent ocular dominance plasticity in V1 into adulthood, suggesting a new and significant role of reelin for cortical plasticity.

It was suggested some 30 years ago that the abnormal laminar position of neurons in *rl*−/− mice does not interfere with the formation of retinotopically ordered afferent connections to V1 (Simmons and Pearlman 1982). These studies were done using single-cell electrophysiology and the layout of retinotopic maps was inferred from histological lesion analysis. Due to the technical limitations at that time, reconstructed maps had a rather low spatial resolution and experiments in individual animals could not reveal maps from the entire V1. Here we used in vivo optical imaging of intrinsic signals, which provides qualitatively and quantitatively unbiased measurements of cortical responses to visual stimulation with high spatial resolution in every mouse (Kalatsky and Stryker 2003; Cang et al. 2005). In agreement with the previous estimations, reelin deficiency did not seem to affect V1-retinotopic organization. In addition, our data provide information about the size, retinotopic map quality and magnitude of stimulus-induced V1-activation in *rl*−/− mice and their WT littermates. Interestingly, none of the measured parameters was different between the genotypes. This finding is in agreement with a recent study imaging the primary somatosensory cortex of *rl*−/− mice, in which similar somatotopic maps were observed in the two genotypes (Guy et al. 2014). Taken together, these studies emphasize the astonishing ability of neuronal circuits to wire up despite a severely compromised positioning of their individual cellular members.

We further observed that visual capabilities of *rl*−/− mice tested in the optomotor setup were indistinguishable from WT mice, and values of visual acuity, contrast sensitivity and temporal resolution of the optomotor reflex were as previously reported for C57Bl/6 mice (Prusky et al. 2004). It is known that the optokinetic reflex is mediated by subcortical pathways (Giolli et al. 2005), so that visual capabilities measured by optomotry mainly reflect the properties of the retinal ganglion cells that project to these subcortical structures (Douglas et al. 2005). Together with previous functional studies, in which grating sensitivity was not different between WT and *rl*−/− mice (Sinex et al. 1979) and anatomical studies showing that reelin is not required for subnucleus-specific targeting of image-forming retinal ganglion cells projecting to the dorsal LGN (Su et al. 2011), our data thus confirm that the functionality of subcortical structures crucial for tracking responses is preserved in *rl*−/− mice. This is especially important given the fact that reelin is expressed in developing subcortical regions (Alcantara et al. 1998), in which its absence causes subtle structural changes such as cytoarchitectural and myeloarchitectural disorganization in the superior colliculus (Baba et al. 2007), or pathologically scattered neuronal populations in subdivisions of the facial nerve nucleus or the cochlear nucleus (Martin 1981; Goffinet 1984a).

Having observed normal basic visual capabilities in *rl*−/− mice we next examined cortex-dependent visual improvement after MD. MD and daily training in an optomotor setup lead to maximal increases in the thresholds of the optomotor reflex through the non-deprived eye and this increase depends on the cortex (Prusky et al. 2006). Interestingly, experience-enabled improvements were similar in *rl*−/− and WT mice, suggesting that the disorganized *rl*−/− cortex was still able to support experience-induced changes. Our results thus suggest that not only subcortical pathways, responsible for the tracking reflex, but also cortical processing required for sensory learning is functional in *rl*−/− mice.

To examine even more elaborate visual processing and learning in *rl*−/− mice, we measured orientation discrimination using the visual water task (Prusky et al. 2000), which is based on reinforcement learning and allows for measurement of perceptual thresholds of the tested animals. Interestingly, *rl*−/− mice learned and remembered the visual discrimination task as well as their WT littermates. This is surprising given the reported hippocampal abnormalities (D’Arcangelo 2005; Boyle et al. 2011) and recent studies showing that *rl*−/− mice exhibit poor active avoidance tests (Goldowitz and Koch 1986; Marrone et al. 2006). However, *rl*−/− mice were perceptually compromised in this task and needed a much larger orientation contrast for a correct behavioral decision compared to WT-mice. Reduced orientation discrimination ability of *rl*−/− mice could be due to a lower number of oriented neurons in V1. In fact, an early electrophysiological study has shown that *rl*−/− mice had a lower overall percentage of oriented cells (Drager 1981). Furthermore, orientation discrimination may need orientation-specific interactions within V1. It is known that layer II/III pyramidal cells of mouse V1 preferentially synapse on other II/III neurons with the same stimulus orientation in the microcircuit (Ko et al. 2011). This feature-selective local connectivity is formed after eye opening and perhaps the disorganized laminar positioning of *rl*−/− neurons makes it more difficult to form the same orientation-selective local wiring as in WT mice (Ko et al. 2011, 2013). It has recently been shown that clonally related visual cortical neurons show similar orientation selectivity and that the functional similarity between sister neurons needs gap junctional coupling during the first postnatal week (Li et al. 2012). An impaired formation of gap junctions in *rl*−/− mice could therefore also underlie the observed phenotype. Alternatively, reelin might be needed for the maturation of neuronal circuits (Ko et al. 2013), so that *rl*−/− mice preserve immature V1 circuits into adulthood with a reduced percentage of orientation-selective cells and a resulting reduced orientation discrimination ability. In fact, the latter possibility fits well with our imaging data showing preserved juvenile OD plasticity into adulthood in *rl*−/− mice.

Studies in mice have shown that OD plasticity in the binocular region of V1 depends on both age (Espinosa and Stryker 2012; Levelt and Hübener 2012) and raising conditions (Greifzu et al. 2014). In standard cage-raised mice, OD plasticity is most pronounced in young animals, reduced, yet present, during adolescence, and absent in animals older than P110 (Lehmann and Löwel 2008; Sato and Stryker 2008). While the WT mice of the present study did not show OD plasticity beyond P110, surprisingly, the P140 *rl*−/− mice continued to show very pronounced OD shifts towards the open eye after MD. In addition, the mechanism underlying OD plasticity in *rl*−/− mice was different from WT: in adult *rl*−/− mice, OD shifts were mediated by a reduction in deprived eye responses in V1. Since such strong OD shifts and reductions in closed eye responses are usually only observed in juvenile, critical period mice (Espinosa and Stryker 2012), our findings suggest that adult *rl*−/− mice preserved a juvenile type of OD plasticity into late adulthood.

Reelin has been suggested to play a significant role in maintenance of neuronal circuits and ongoing plasticity during adulthood (Stranahan et al. 2013). A recent study investigating cortico-striatal plasticity showed that *rl*−/− mice preferentially exhibited LTP following high-frequency stimulation whereas WT littermates expressed LTD under the same conditions (Marrone et al. 2006). Moreover, the number of PV^+^-GABAergic interneurons was reduced in the striatum of *rl*−/− mice, and blocking GABA re-uptake reinstated normal cortico-striatal plasticity, suggesting that reduced GABAergic neurotransmission in *rl*−/− mice had mediated the observed effects. GABAergic circuits play an important role in both juvenile and adult experience-dependent plasticity in the visual cortex (Kirkwood et al. 1995; Hensch et al. 1998; Huang et al. 1999). In adult rodents, experimental reduction of the inhibitory tone can partially restore OD plasticity (Maya Vetencourt et al. 2008a; Harauzov et al. 2010; Morishita et al. 2010), suggesting that developmental strengthening of local inhibitory circuits is limiting adult plasticity. Thus, it was possible that the preserved juvenile OD plasticity in adult *rl*−/− mice was the result of a reduction in inhibitory tone, as in the striatum. We examined this possibility in vivo by increasing GABAergic transmission with diazepam, an allosteric agonist of the GABA_A_ receptor, during the 7 days of MD. Diazepam treatment completely and reliably blocked OD plasticity in WT but not in *rl*−/− mice. Additionally, while immunofluorescence analyses showed a slightly reduced number of PV^+^ cells in *rl*−/− V1, the values were not significantly different from WT. Taken together, the preservation of juvenile OD plasticity in *rl*−/− mice is not likely mediated by changes in inhibitory tone, suggesting that other functional and/or morphological changes might be responsible for enhanced and prolonged plasticity in *rl*−/− mice. During adulthood, reelin binding to its receptors was shown to modulate NMDA receptor signaling altering synaptic plasticity (Chen et al. 2005; Herz and Chen 2006), thus it is possible that enhanced and prolonged visual cortex plasticity in *rl*−/− mice is due to modified glutamatergic signaling. Alternatively reelin promotes maturation of neuronal processes (D’Arcangelo 2005) so that reelin deficiency might preserve a more juvenile brain.

In summary, we could disentangle remarkable capacities of sensory stimulus representation, processing and learning in a highly disturbed cortex, which underscore the striking plasticity of neocortical circuits. In addition*, rl*−/− mice display an unexpected increased plasticity of their V1-circuits, the precise molecular and physiological mechanisms of which need to be unraveled in future studies.
